# Anti-Arrhythmic Effects of Heart Failure Guideline-Directed Medical Therapy and Their Role in the Prevention of Sudden Cardiac Death: From Beta-Blockers to Sodium-Glucose Cotransporter 2 Inhibitors and Beyond

**DOI:** 10.3390/jcm13051316

**Published:** 2024-02-26

**Authors:** Wael Zaher, Domenico Giovanni Della Rocca, Luigi Pannone, Serge Boveda, Carlo de Asmundis, Gian-Battista Chierchia, Antonio Sorgente

**Affiliations:** 1 Department of Cardiology, Centre Hospitalier EpiCURA, Route de Mons 63, 7301 Hornu, Belgium; wael.zaher@ulb.be; 2 Heart Rhythm Management Centre, Postgraduate Program in Cardiac Electrophysiology and Pacing, Universitair Ziekenhuis Brussel-Vrije Universiteit Brussel, European Reference Networks Guard-Heart, Laarbeeklan 101, Jette, 1090 Brussels, Belgium; domenicodellarocca@hotmail.it (D.G.D.R.); lui.pannone@gmail.com (L.P.); carlodeasmundis@me.com (C.d.A.); jeanbaptiste.chierchia@uzbrussel.be (G.-B.C.); 3 Heart Rhythm Management Department, Clinique Pasteur, 31076 Toulouse, France; sboveda@clinique-pasteur.com

**Keywords:** heart failure with reduced ejection fraction, sudden cardiac death, ventricular arrhythmias, guideline-directed medical therapy, pharmacological management

## Abstract

Sudden cardiac death (SCD) accounts for a substantial proportion of mortality in heart failure with reduced ejection fraction (HFrEF), frequently triggered by ventricular arrhythmias (VA). This review aims to analyze the pathophysiological mechanisms underlying VA and SCD in HFrEF and evaluate the effectiveness of guideline-directed medical therapy (GDMT) in reducing SCD. Beta-blockers, angiotensin receptor–neprilysin inhibitors, and mineralocorticoid receptor antagonists have shown significant efficacy in reducing SCD risk. While angiotensin-converting enzyme inhibitors and angiotensin receptor blockers exert beneficial impacts on the renin-angiotensin-aldosterone system, their direct role in SCD prevention remains less clear. Emerging treatments like sodium-glucose cotransporter 2 inhibitors show promise but necessitate further research for conclusive evidence. The favorable outcomes of those molecules on VA are notably attributable to sympathetic nervous system modulation, structural remodeling attenuation, and ion channel stabilization. A multidimensional pharmacological approach targeting those pathophysiological mechanisms offers a complete and synergy approach to reducing SCD risk, thereby highlighting the importance of optimizing GDMT for HFrEF. The current landscape of HFrEF pharmacotherapy is evolving, with ongoing research needed to clarify the full extent of the anti-arrhythmic benefits offered by both existing and new treatments.

## 1. Introduction

Heart failure (HF), and, more specifically, heart failure with reduced ejection fraction (HFrEF), stands as a public health challenge characterized by substantial morbidity and mortality, notably attributed to sudden cardiac death (SCD). The incidence of SCD in the HF population is fivefold greater than in the general population, accounting for 40–50% of all deaths, which is a considerable proportion of potentially preventable deaths in this population [1]. The association between SCD and HF is predominantly mediated by ventricular arrhythmias (VA), resulting from the complex interplay between the structurally altered cardiac substrate in HF and various environmental triggers [2,3].

Guideline-directed medical therapy (GDMT) for HFrEF is founded on a cornerstone of four distinct classes of medications: beta-blockers (BB), renin-angiotensin-aldosterone system (RAAS) inhibitors, mineralocorticoid receptor antagonists (MRA), and sodium-glucose cotransporter 2 inhibitors (SGLT2i) [4]. Each of these pharmacological classes has demonstrated significant efficacy in reducing major cardiac events in HFrEF patients, including, for some, reduction in the incidence of SCD [5,6]. In this comprehensive review, we delve into the pathophysiological mechanisms underlying VA and SCD within the context of HF, and we undertake an in-depth analysis of the beneficial effects conferred by each of these medication classes in preventing SCD in this population.

## 2. Pathophysiology of Ventricular Arrhythmia and Sudden Cardiac Death

The pathophysiology of SCD due to VA in the context of HF is multifaceted. It involves an intricate interplay among transient factors or events that serve as triggers (e.g., ischemic episodes, metabolic disturbances, electrolyte imbalances, fluctuations in sympathetic nervous system activity), occurring in the presence of a myocardial substrate predisposed to arrhythmias [3,7].

Three primary electrophysiological mechanisms lead to VA within this context: (1) Inappropriately Increased Automaticity: This mechanism involves certain ventricular myocyte regions exhibiting increased automaticity, thereby initiating spontaneous action potentials without external stimulation, contributing to the onset of arrhythmias. (2) Triggered Activity: Triggered activity can manifest as early afterdepolarizations (occurring late in phase 2 or early in phase 3 of the action potential) or delayed afterdepolarizations (occurring after complete repolarization). These afterdepolarizations can reach the threshold potential necessary for the activation of ion channels, ultimately leading to arrhythmic events. Triggered activity typically follows a preceding impulse and is not a self-generating rhythm. (3) Reentry: This involves the circulation of electrical impulses around a barrier, often anatomical, such as scar tissue or fibrosis, perpetuating a continuous myocardial excitation, leading to sustained arrhythmias. It is noteworthy that these three mechanisms can coexist; arrhythmias may initiate through triggered activity and transition to reentrant patterns [7,8,9].

The pathophysiological changes in HF create an environment that favors these VA mechanisms. HF is characterized by cardiac remodeling, which includes ventricular enlargement, hypertrophy, and fibrosis [10]. Fibrosis may result from myocardial infarctions or from other structural cardiac disorders such as hypertrophic cardiomyopathy and arrhythmogenic cardiomyopathy. Factors like TGF-β1, endothelin-1, and Angiotensin II induce fibrosis, perturbing electrical excitation and repolarization [11]. Regions of fibrosis facilitate reentrant tachycardias by disrupting myocardial electrical impulses and fostering areas of slow conduction [12].

The nervous system and the RAAS, which are both markedly activated in the setting of HFrEF due to ventricular dysfunction, are significantly implicated in arrhythmogenesis. Angiotensin II and Aldosterone, acting through RAAS, induce vasoconstriction, increasing the afterload and remodeling, and stimulate fibroblasts’ activity, leading to interstitial fibrosis and scar formation [10,13]. At the cellular level, angiotensin II initiates multiple signaling pathways that induce electrical remodeling, notably through alterations in the sodium current [14] and the destabilization of Kv4.3 messenger RNA [15], leading to increased Ca^2+^/calmodulin-dependent protein kinase II (CaMKII) activity, perturbing calcium homeostasis, and predisposing to VA [16,17,18]. Moreover, Angiotensin II stimulates the sympathetic nervous system, contributing to VA through an increased sympathetic tone. Elevated levels of norepinephrine raise the afterload, thus increasing the myocardial oxygen demand and promoting cardiac remodeling, dilatation, and fibrosis [10]. Such structural alterations create a favorable environment for arrhythmogenesis. Additionally, sympathetic activation induces electrophysiological alterations, disrupting sodium and calcium channel currents and prolonging action’s potential duration, ultimately predisposing the myocardium to early depolarizations [19]. For example, chronic adrenergic activation destabilizes Ryanodine receptors 2 (RyR2) channels, which are critical Ca^2+^ release channels located on the sarcoplasmic reticulum, playing a central role in the excitation-contraction coupling in the heart. Dysfunctional RyR2 channels lead to calcium leak and elevated cytoplasmic calcium levels via CaMKII, impairing cardiac function and exacerbating arrhythmogenesis through electrical instability, delayed afterdepolarizations, and triggered arrhythmias [18,19].

**Figure 1:**
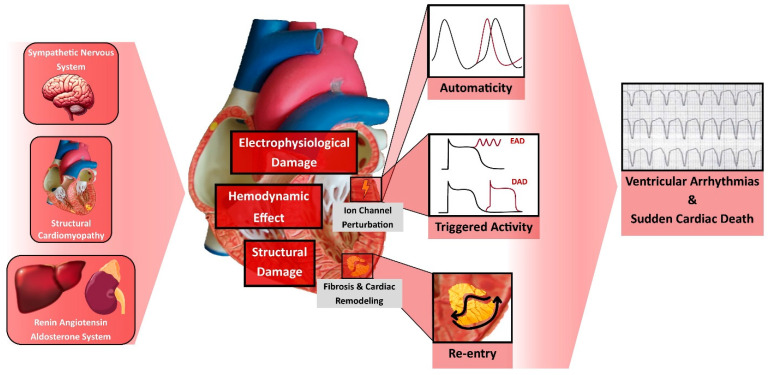
Pathophysiology of Ventricular Arrhythmia and Sudden Cardiac Death in Heart Failure. DAD, Delayed Afterdepolarization; EAD, Early Afterdepolarization.

All this intricate electrical and structural remodeling in HFrEF leads to arrhythmogenesis and SCD (Figure 1). This highlights the importance of the GDMT for HFrEF in mitigating the risk of SCD in this population.

## 3. Beta-Blockers

β-adrenergic receptors are expressed on cardiomyocytes. The activation of the cardiac sympathetic system by the binding of norepinephrine and epinephrine to these receptors initiates a cascade of intracellular reactions involving the cAMP/PKA signaling pathway [20]. This signaling pathway affects several key targets, including RyR2, L-type calcium channels, phospholamban, and others [19,20]. The net result of these signaling events is the enhancement of various aspects of cardiac function: an increased heart rate (chronotropy), an accelerated conduction velocity (dromotropy), a heightened force of contraction (inotropy), and an improved speed of relaxation (lusitropy) [21]. All these downstream effects of β-adrenergic stimulation facilitate the development of ectopic activity, including early afterdepolarizations and delayed afterdepolarizations, as well as functional reentry through action potential duration shortening, effective refractory period reduction, and conduction alterations [21]. Consequently, excessive β-adrenergic stimulation is associated with arrhythmias.

Three subtypes of β-receptors are known, with β1-receptors being predominantly localized in the cardiac tissue. The classes of BB are diverse, encompassing non-selective β-adrenergic antagonists (e.g., nadolol, propranolol), β1-selective adrenergic antagonists (e.g., acebutolol, atenolol, esmolol, metoprolol), and β-adrenergic antagonists with additional cardiovascular effects (e.g., carvedilol, labetalol, nebivolol), which may exhibit vasodilatory, anti-inflammatory, and antioxidant properties [22]. BB attenuate the sympathetic tone and sympathetically mediated triggers [22]. They mitigate automaticity by prolonging the sinus node cycle length and decelerating the atrioventricular conduction velocity by limiting calcium entry via catecholamine-dependent channels [21]. Moreover, BB have been shown to possess antifibrotic properties, improve the left ventricular (LV) function, reduce the LV diameter, and decrease the LV mass, outcomes that are favorable in reducing SCD in HF [21].

Multiple studies have demonstrated the efficacy of BB in preventing arrhythmias and SCD in the context of HF. CIBIS II investigated the impact of Bisoprolol in HFrEF and demonstrated a significant mortality benefit, with a 42% reduction in SCD compared to the placebo group [23]. Metoprolol in HFrEF showed a similar and significant reduction in SCD in the MERIT-HF trial [24]. In the SENIORS trial, evaluating Nebivolol in an elderly HFrEF population, the SCD rate was significantly lower in the nebivolol-treated group [25]. The COPERNICUS trial in 2001, studying Carvedilol, showed a mortality benefit, although explicit data on SCD were not presented [26]. Additionally, several studies have indicated a significant reduction in shocks in patients wearing implantable cardioverter–defibrillators (ICD) when treated with BB compared to those without [27]. Meta-analyses have confirmed the benefit of BB on both mortality and SCD, without revealing statistically significant differences among the various types of BB [28,29,30]. Recent evidence, however, indicates a potential superiority of non-selective beta-blockers in antiarrhythmic effects. Specifically, Propranolol has been demonstrated to be superior to Metoprolol in managing electrical storms in ICD patients [31]. Carvedilol has also been associated with a substantial decrease in VA risk, compared to Metoprolol [32]. Further robust data are needed to confirm these findings.

In conclusion, BB substantially reduce proarrhythmic risk by inhibiting sympathetically mediated triggers, reducing functional reentrant substrates, and slowing the sinus node and atrioventricular nodal rates. The evidence supporting their use in HFrEF is robust and emphasizes their critical role in mitigating arrhythmias and SCD in this patient population.

## 4. Angiotensin-Converting Enzyme Inhibitor/Angiotensin Receptor Blocker

The RAAS exerts a critical influence on the pathophysiology of HFrEF by contributing to cardiac remodeling, interstitial fibrosis, and perturbations in various ionic currents, which collectively enhance arrhythmogenesis. The inhibition of the RAAS is principally achieved through two pharmacological classes: angiotensin-converting enzyme inhibitors (ACEi) and angiotensin II receptor blockers (ARB). ACEi act by inhibiting the conversion of angiotensin I to angiotensin II, while ARB selectively antagonize the angiotensin II type 1 receptors [33]. This blockage results in several beneficial effects, including the attenuation of ventricular remodeling, vasodilatation, a reduction in neurohormonal agents, and a decrease in the sympathetic tone and circulating catecholamines, along with favorable effects on ionic currents [10,34].

Several pivotal trials have explored the efficacy of RAAS inhibitors in the context of HFrEF. The CONSENSUS study, assessing the use of Enalapril in HFrEF, demonstrated a significant reduction in mortality, although it did not find a difference in SCD [35]. Similar results were found in the SOLVD-Prevention study [36]. In the ELITE trial, which compared Losartan to Captopril, Losartan was associated with a lower mortality, primarily driven by a lower incidence of SCD [37]. However, these results were not replicated in the ELITE II trial, where no significant differences were observed in either mortality or SCD [38]. Trials such as CHARM (exploring Candesartan) [39] and Val-HEFT (exploring Valsartan) [40] did not specifically monitor SCD as an endpoint. In patients wearing ICD, ACEi/ARB therapy has been associated with an improved freedom from shocks [41,42]. A large retrospective registry by Schupp et al. [43] indicated that ACEI/ARB treatment is associated with reduced all-cause mortality in patients who survived episodes of VA. Nonetheless, meta-analyses have shown a lack of a consistent efficacy of ACEi/ARBs in reducing the risk of SCD and VA, despite demonstrating benefits in overall mortality and hospitalization rates [30,44,45,46]. The exception appears to be in the context following acute myocardial infarction, where ACEi have shown a 20% reduction in SCD [47].

In conclusion, although ACEi and ARB have demonstrated favorable effects on the RAAS and have robust data supporting their beneficial impact on overall outcomes in HFrEF, their role in reducing the risk of SCD has not been definitively established, as underscored by multiple meta-analyses.

## 5. Angiotensin Receptor–Neprilysin Inhibitor

The Angiotensin Receptor–Neprilysin Inhibitor (ARNi) combines two active components: Valsartan, an ARB, and Sacubitril, a Neprilysin Inhibitor [48]. The molecular mechanisms underlying the anti-arrhythmic effects of ARNi are not yet fully elucidated and remain speculative to some extent. The mechanism of action encompasses effects on the RAAS via the ARB component and additional effects attributable to the Neprilysin Inhibitor, Sacubitril. At the electrophysiological level, ARNi exerts an influence on ionic currents in cardiomyocytes. Chang et al. [49] observed that ARNi had the potential to up-regulate the expression of potassium channel proteins, including KCNH2, KCNE1, and KCNE2. The consequent shortening of the action potential duration potentially ameliorates ventricular arrhythmogenicity, especially in the context of HF induced by myocardial infarction. Moreover, ARNi also appears to have a favorable effect on calcium homeostasis by downregulating the expression of CaMKII and mitigating diastolic calcium leak arising from dysfunctional RyR2 [50,51]. Additionally, neprilysin inhibition by ARNi contributes to elevated circulating levels of natriuretic peptides, which exert various cardioprotective effects countering the detrimental effects of the RAAS and sympathetic nervous system activation [52]. Natriuretic peptides increase the intracellular levels of cyclic guanosine monophosphate and its downstream effector molecule protein kinase G. This cascade leads to vasodilation, natriuresis, the inhibition of the RAAS, and sympathetic systems [52]. Furthermore, natriuretic peptides exert anti-inflammatory, anti-apoptotic, anti-hypertrophic, and anti-fibrotic effects on the myocardium [53,54,55]. ARNi has also been shown to significantly reduce biomarkers associated with profibrotic signaling [56].

Multiple studies and trials corroborate the beneficial effects of ARNi on VA and ICD therapy [57,58]. Post hoc analyses of the PARADIGM-HF trial, investigating ARNi in HFrEF, showed a significant reduction in VA and the risk of SCD [59,60]. Liu et al. [61] found in their meta-analyses that although ARNi did not affect the incidence of VA, it did significantly reduce the risk of SCD in heart failure patients. Another meta-analysis by Pozzi et al. [62] demonstrated a significant reduction in the burden of VA and ICD shock when comparing ARNi to ACEi/ARB therapy. Fernandes et al. [63] reported a significant reduction in SCD, VA, and appropriate ICD therapy with ARNi. Major studies and meta-analyses regarding the effect of ARNi on VA and SCD are summarized in Table 1.

In conclusion, ARNi therapy appears to manifest favorable effects via multiple mechanisms, including vasodilation, the attenuation of sympathetic activation, the reduction in myocardial wall stretch and fibrosis, and modulatory impacts on ion channels such as potassium channels, RyR2, and the CaMKII pathway. Meta-analyses confirm ARNi’s efficacy in reducing both VA and SCD, yet further investigation is needed to fully understand the precise molecular mechanisms.

## 6. Mineralocorticoid Receptor Antagonists 

MRA provide a complementary approach to the neurohormonal suppression of the RAAS by targeting the aldosterone receptor [66]. These agents achieve a series of beneficial cardiovascular outcomes, including the prevention of the electrical remodeling of cardiac tissue [67,68], the attenuation of myocardial fibrosis and ventricular remodeling [69,70], a decrease in sympathetic activation [71], and beneficial effects on endothelial vasomotor dysfunction [72]. Through these several mechanisms, MRA have been shown to prevent SCD.

The RALES trial, which investigated the use of Spironolactone in patients with HFrEF, reported a significant reduction in both overall mortality and cardiac-specific mortality. It showed a 29% reduction in the risk of SCD [73]. Similarly, the EPHESUS trial focusing on Eplerenone demonstrated a significant reduction in all-cause mortality, cardiovascular-related death, and the risk of cardiovascular-related death or hospitalization. Notably, it also showed a significant reduction in the incidence of SCD [74]. Numerous meta-analyses have consistently shown a clear benefit for MRA in reducing SCD, reinforcing their critical role in managing patients with HFrEF [30,75,76,77].

## 7. Sodium-Glucose Cotransporter 2 Inhibitors

SGLT2i have emerged as a recent addition to the GDMT for HFrEF [4]. They primarily function by the selective inhibition of the SGLT2 receptor in the renal proximal tubules, conferring a variety of metabolic and cardiovascular benefits, including a reduction in cardiovascular mortality in patients with HFrEF [78].

The specific anti-arrhythmic mechanisms of SGLT2i are not yet completely understood, but several plausible pathways have been proposed. SGLT2i have been shown to exhibit anti-oxidative and anti-inflammatory properties by promoting multiple anti-oxidant and anti-inflammatory signaling pathways [79,80]. For instance, they activate AMP-activated protein kinase [81,82], attenuate the nucleotide-binding domain-like receptor protein 3 (NLRP3) inflammasome [83], and reduce the expression of inflammatory factors. This reduction in inflammation and oxidative stress may lead to anti-fibrotic and anti-remodeling properties, thereby decreasing the arrhythmic substrate. Data have indicated a reversal in cardiac volumes, a decrease in LV mass, and an improvement of LV function following SGLT2i treatment [84,85,86]. These positive changes have also been observed in the right ventricle [87]. Another anti-arrhythmic mechanism of SGLT2i is through ionic channels modulation. SGLT2i may modulate ionic channels by restoring intracellular Ca^2+^ and Na^+^ homeostasis. HF is characterized by cellular Na+ overload, often mediated by the upregulation of the Na^+^/H^+^ exchanger 1 (NHE1), contributing to cytosolic Ca^2+^ overload, thus increasing arrhythmogenic risk [88,89]. SGLT2i can mitigate this risk by directly reducing Na^+^ overload through the inhibition of the late sodium current (INaL) and NHE1 [90,91]. Moreover, they may indirectly modulate CaMKII activity, thereby reducing calcium leakage from the sarcoplasmic reticulum and diminishing arrhythmic triggers like afterdepolarizations [92]. SGLT2i have also been shown to normalize the QT interval and QT dispersion, potentially reducing arrhythmogenic risks [93,94]. Experimental models have demonstrated that SGLT2i reduce ventricular arrhythmia vulnerability following myocardial ischemia [95].

Clinical trials, like DAPA-HF [96] and EMPEROR-Reduced [97], have shown a decrease in cardiovascular-related mortality in HFrEF with Dapagliflozin and Empagliflozin, respectively. Post-hoc analyses have indicated that Dapagliflozin reduces the risk of VA, cardiac arrest, and SCD [98]. Nonetheless, data from several meta-analyses have been inconclusive in showing a significant reduction in SCD or VAs with SGLT2i [99,100,101]. It is worth noting that many of these meta-analyses often included studies with SGLT2i focusing on diabetic patients without the specific consideration of HF. More recently, a meta-analysis by Oates et al. [102] focused specifically on the HF population and showed that SGLT2i therapy is associated with a reduced risk of SCD in patients with HF receiving contemporary medical therapy. The ongoing trial EMPA-ICD, assessing the effect of SGLT2i on VA in patients with type 2 diabetes (T2DM) wearing an ICD, is expected to provide additional data on the anti-arrhythmic effect of SGLT2i [103]. Major studies and meta-analyses regarding the effect of SGLT2i on VA and SCD are summarized in Table 2.

In conclusion, SGLT2i appear to exhibit anti-arrhythmic properties through various pleiotropic mechanisms, including the restoration of calcium and sodium homeostasis, the reversal of cardiac remodeling, and the exertion of antioxidant and anti-inflammatory effects. Although current data trends suggest a reduced incidence of SCD and VA, further well-designed prospective studies are imperative for the definitive validation of these anti-arrhythmic effects.

## 8. Implantable Cardioverter–Defibrillator in Light of New Heart Failure Treatment

ICD therapy is a cornerstone in the management of HFrEF, offering a proven benefit in reducing the risk of SCD. According to the current European Society of Cardiology (ESC) guidelines, ICD implantation is recommended with a class I indication for patients with ischemic HF and a left ventricular ejection fraction (LVEF) <35%, despite medical therapy for 3 months or more, and at a New York Heart Association (NYHA) functional class II or III. For patients with non-ischemic dilated cardiomyopathy, a Class IIa recommendation is provided [7]. Medical therapy includes the four classes of medications (BB, RAAS inhibitor, MRA, SGLT2i), each of which presents a Class I recommendation [4]. Similarly, the American Heart Association/American College of Cardiology/Heart Failure Society of America (AHA/ACC/HFSA) 2022 guidelines also endorse a Class 1 recommendation for both ischemic and non-ischemic patients [105].

These recommendations derive their basis from pivotal trials conducted over two decades ago—notably, MADIT II [106], SCD-HeFT [107], and DEFINITE [108]. In these studies, patient management included BB and RAAS inhibitors, such as ACEi and ARB, with approximately 20% of participants in the SCD-HeFT trial receiving an MRA. The more recent DANISH trial, however, did not demonstrate a significant reduction in all-cause mortality with ICD use, though a reduction in SCD was observed [109]. This outcome may be attributable to the inclusion of an older patient cohort, the extensive use of cardiac resynchronization therapy in both arms, or potentially, better medical management including the broader use of MRA and higher utilization rates of BB and ACEi/ARB compared to earlier trials. The latter highlights the importance of optimal medical therapy.

In the context of today’s GDMT, including newer treatments such as ARNi and SGLT2i with a beneficial anti-arrhythmic profile, the criteria for ICD therapy may merit reconsideration. Prior data, predicated on outdated HFrEF management protocols, may no longer fully apply. Does the current GDMT have a sufficient anti-arrhythmic effect to disturb the benefit–risk balance of primary prevention ICD in HFrEF? Only proper trials could answer this question. Moreover, the timing for ICD implantation, currently recommended after 3 months of medical therapy, may also be challenged. The PROVE-HF trial indicated that within an HFrEF cohort treated with ARNi, 32% of patients exhibited an improvement in LVEF to over 35% at 6 months and 62% at 12 months [110]. Consequently, patients initially eligible for ICD therapy may no longer meet the criteria within this timeframe, suggesting that an ICD implantation at 3 months might be premature in this era of novel HF therapy. This improvement of LVEF with GDMT may play an important role in the burden reduction of VA. For example, Martens et al. [57] reported that patients treated with ARNi exhibiting more left ventricular reverse remodeling (defined by a mean improvement in LVEF of 5%) present a significantly lower burden of VA, compared to those with less reverse remodeling. Besides their direct beneficial effect on structural substrate and electrical remodeling, GDMT’s improvement of LVEF may by itself decrease the arrhythmic burden, due to its hemodynamic benefit with a more favorable neurohormonal profile.

All these considerations question the exact place of ICD in this contemporary era of HF medication. In the absence of appropriate trials that incorporate the latest GDMT, adherence to existing guidelines is obligatory. However, there is a compelling need for updated research to more precisely define the indications and optimal timing for ICD implantation in the current therapeutic landscape.

## 9. Emerging Horizons in Heart Failure Treatment

Vericiguat and Omecamtiv Mecarbil represent newer pharmacological interventions for HFrEF. Vericiguat is a soluble guanylate cyclase (sGC) activator. By modulating the sGC-cyclic guanosine monophosphate pathway, Vericiguat induces vasodilatation, which in turn has beneficial effects on left ventricular afterload and hypertrophy [111,112]. The VICTORIA Study demonstrated that Vericiguat led to a statistically significant reduction in the primary endpoint of death from cardiovascular causes or heart failure hospitalizations. However, data specifically concerning SCD were not provided [113]. In pre-clinical mouse models, Vericiguat demonstrated the potential for anti-arrhythmic properties through favorable effects on ventricular remodeling and ionic currents, notably by inhibiting CaMKII [114]. Further investigations are warranted to validate these preliminary findings on its anti-arrhythmic properties.

Omecamtiv Mecarbil, on the other hand, was evaluated in the GALACTIC-HF trial in 2020 and showed a significant but modest lowering of the incidence of the primary composite outcome of heart failure hospitalization or death from cardiovascular causes [115]. The incidence of VA in the Omecamtiv Mecarbil cohort was analogous to that of the placebo group. Given these results, the anti-arrhythmic potential of Omecamtiv Mecarbil remains inconclusive at present.

Another drug worth mentioning is Glucagon-like peptide 1 receptor agonists (GLP-1 RA), which represent a relatively recent class of drugs introduced for the treatment of T2DM, exhibiting a range of diverse effects—notably, favorable metabolic outcomes [116]. Major trials investigating GLP-1 RA in T2DM have shown a reduction in major adverse cardiovascular events [117,118,119]. Regarding the HFrEF population specifically, two pivotal trials warrant mention. The FIGHT study, evaluating Liraglutide in HFrEF, reported no significant benefits, including in LVEF [120]. A post hoc analysis showed an excess risk of arrhythmias and HF events [121]. Furthermore, the LIVE study associated Liraglutide treatment with an increase in the heart rate and serious cardiac adverse events, including VT [122]. This brings concerns about the risk of VAs and SCD in HFrEF patients. Multiple meta-analyses regarding the T2DM population did not reveal an increased risk for VAs or SCD with GLP-1 RA use [123,124]. The overall impact of GLP-1 RAs on VA and SCD in the HFrEF population remains inconclusive, as does their general effect in HFrEF management. Nonetheless, their utility in heart failure with preserved ejection fraction (HFpEF) appears more promising, attributed to their broad spectrum of effects, including weight loss [116], improved diastolic function [125], and mitochondrial function enhancements [126]. This is supported by the STEP-HFpEF trial, endorsing the use of Semaglutide in HFpEF patients with an obesity phenotype [127].

**Figure 2:**
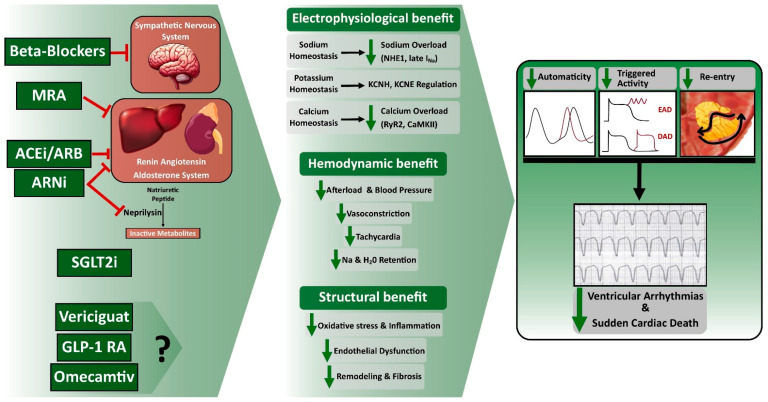
Effects of Guideline-directed medical therapy on Ventricular Arrhythmia and Sudden Cardiac Death. ACEi, Angiotensin-Converting Enzyme Inhibitors; ARB, Angiotensin II Receptor Blocker; ARNi, Angiotensin Receptor–Neprilysin Inhibitors; DAD, Delayed Afterdepolarization; EAD, Early Afterdepolarization; GLP-1 RA, Glucagon-like peptide 1 receptor agonists; MRA, Mineralocorticoid Receptor Antagonist; SGLT2i, Sodium-Glucose Cotransporter 2 Inhibitor.

All these new drugs offer innovative therapeutic approaches for HF, though the data on their anti-arrhythmic effects are limited and inconclusive (Figure 2). The outcomes of these initial trials signal the need for more targeted investigations, especially about potential anti-arrhythmic properties and their impact on SCD and VA.

## 10. Conclusions

In conclusion, SCD constitutes a critical contributor to mortality among patients with HFrEF, predominantly due to malignant VA. The optimization of GDMT serves as an effective strategy to mitigating the incidence of SCD within this population. Therapeutic interventions primarily target key systems such as the RAAS and the sympathetic nervous system, preventing both structural and electrical remodeling of the myocardium.

Despite their favorable modulatory effects on RAAS, ACEi and ARB have not been conclusively associated with a reduction in either SCD or VA. Robust evidence supports the utility of BB, ARNi, and MRA in reducing the risk of SCD. Furthermore, SGLT2i offer promising preliminary data, necessitating further well-designed prospective studies for confirmation of their anti-arrhythmic effects. Through the employment of different complementary mechanisms of action, those molecules act in synergy, highlighting the importance of optimizing GDMT to reduce arrhythmic risk in HFrEF.

Overall, the landscape of pharmacological interventions for HFrEF is progressively expanding, with novel agents undergoing evaluation. Further research is imperative for the unequivocal delineation of these agents’ impact on SCD and VA, thus augmenting our arsenal in the management of HFrEF.

## Figures and Tables

**Table 1:** Major studies and meta-analyses regarding effect of ARNi on ventricular arrythmia and sudden cardiac death.

Authors Journal Year	Type of Study Intervention	No. of Patients in the Population	Effect on VA and SCD
Martens, et al. Clin Res Cardiol. 2019 [57].	Retrospective, cohort Pre- vs. Post-ARNi initiation	151 HFrEF with ICD	VA reduction (51 vs. 14; *p* < 0.001) ICD shock reduction (16 vs. 6; *p* < 0.001)
De Diego, et al. Heart Rhythm. 2018 [58].	Prospective, cohort ARNi vs. ACEi/ARB	240 HFrEF with ICD	VA and ICD shock reduction (0.8% vs. 6.7%; *p* < 0.02)
Russo, et al. J Clin Med. 2020 [64].	Prospective, cohort ARNi	167 HFrEF with ICD	VA reduction (15 vs. 4; *p* = 0.03) ICD shock reduction (13 vs. 3; *p* = 0.02)
Rohde et al. JACC Heart Fail. 2020 [59].	RCT—post hoc analysis ARNi vs. ACEi	8399 HFrEF	SCD reduction in the ICD group (HR 0.49; 95% CI 0.25–0.99) and non-ICD group (HR 0.81; 95% CI 0.67–0.98)
Curtain, et al. Eur J Heart Fail. 2022 [60].	RCT—post hoc analysis ARNi vs. ACEi	8399 HFrEF	VA reduction (HR 0.76; 95% CI 0.62–0.95)
Fernandes, et al. Heart Rhythm O2. 2021 [63].	Meta-analysis ARNi vs. ACEi/ARB	11,204 HFrEF	SCD reduction (OR 0.78; 95% CI 0.63–0.96) VA reduction (OR 0.45; 95% CI 0.25–0.79) Higher BiV Pacing (*p* < 0.0001)
Liu, et al. Front Cardiovasc Med. 2022 [61].	Meta-analysis ARNi vs. ACEi/ARB/Placebo	18,500 HFrEF or HFpEF	No VA reduction (RR 0.86; 95% CI 0.68–1.10) SCD reduction (RR 0.79; 95% CI 0.70–0.90)
Mujadzic, et al. J Innoc Card Rhythm Mang. 2022 [65].	Meta-analysis ARNi vs. ACEi/ARB/Placebo	18,548 HFrEF or HFpEF	VA & SCD reduction (OR 0.71; 95% CI 0.54–0.93) ICD shock reduction (OR 0.23; 95% CI 0.11–0.47)
Pozzi, et al. Heart Fail Rev. 2023 [62].	Meta-analysis ARNi vs. ACEi/ARB	8837 HFrEF	VA reduction (OR 0.78; 95% CI 0.63–0.96 for RCT and RR 0.62; 95% CI 0.53–0.72 for observational studies) ICD shock reduction (RR 0.24; 95% CI 0.12–0.24)

ACEi, Angiotensin-Converting Enzyme Inhibitor; ARB, Angiotensin II Receptor Blocker; ARNi, Angiotensin Receptor–Neprilysin Inhibitor; BiV, Biventricular; HFpEF, Heart Failure with Preserved Ejection Fraction; HFrEF, Heart Failure with Reduced Ejection Fraction; ICD, Implantable Cardioverter–Defibrillator; RCT, Randomized Controlled Trial; SCD, Sudden Cardiac Death; VA, Ventricular Arrhythmia.

**Table 2:** Major studies and meta-analyses regarding the effect of SGLT2i on ventricular arrhythmia and sudden cardiac death.

Authors Journal Year	Type of Study Intervention	No. of Patients in the Population	Effect on VA and SCD
Curtain, et al. Eur Heart J. 2021 [98].	RCT—post hoc analysis Dapaglifozin vs. Placebo	4744 HFrEF	Reduction in the composite outcome of VA and SCD (HR 0.79; 95% CI 0.63–0.99)
Fernandes, et al. Heart Rhythm. 2021 [99].	Meta-analysis SGLT2i vs. Placebo	63,166 T2DM or HF	SCD reduction (HR 0.72; 95% CI 0.54–0.97) No difference in VA
Li, et al. Cardiovasc Diabetol. 2021 [104].	Meta-analysis SGLT2i vs. Placebo	52,115 T2DM or CKD or HF	VA reduction (RR 0.73; 95% CI 0.53–0.99)
Sfairopoulos et al. Europace. 2022 [100].	Meta-analysis SGLT2i vs. Placebo	55,590 T2DM or CKD or HF	No VA reduction (RR 0.84; 95% CI 0.66–1.06) No SCD reduction (RR 0.74; 95% CI 0.50–1.08)
Yin et al. Front Cardiovasc Med. 2022 [101].	Meta-analysis SGLT2i vs. Placebo	10,344 HFrEF or HFpEF	No VA reduction (VT: RR 0.90; 95% CI 0.44–1.82; VF: RR 1.40; 95% CI 0.73–2.67)
Oates, et al. J Cardiovasc Electrophysiol. 2023 [102].	Meta-analysis SGLT2i vs. Placebo	10,796 HFrEF or HFpEF	SCD reduction (RR 0.68; 95% CI 0.48–0.95) No VA reduction (RR 1.03; 95% CI 0.83–1.29)

CKD, Chronic Kidney Disease; HF, Heart Failure; HFpEF, Heart Failure with preserved ejection fraction; HFrEF, Heart Failure with reduced ejection fraction; RCT, Randomized Controlled Trial; SCD, Sudden Cardiac Death; SGLT2i, Sodium-Glucose Cotransporter 2 Inhibitor; T2DM, Type 2 Diabetes; VA, Ventricular Arrhythmia; VF, Ventricular Fibrillation; VT, Ventricular Tachycardia.

## Data Availability

No new data were created or analyzed in this study. Data sharing is not applicable to this article.
